# Improved Efficiency of Perovskite Light-Emitting Diodes Using a Three-Step Spin-Coated CH_3_NH_3_PbBr_3_ Emitter and a PEDOT:PSS/MoO_3_-Ammonia Composite Hole Transport Layer

**DOI:** 10.3390/mi10070459

**Published:** 2019-07-07

**Authors:** Yuanming Zhou, Sijiong Mei, Dongwei Sun, Neng Liu, Wuxing Shi, Jiahuan Feng, Fei Mei, Jinxia Xu, Yan Jiang, Xianan Cao

**Affiliations:** 1 Hubei Key Laboratory for High-efficiency Use of Solar Energy and Operation Control of Energy Storage System, Hubei University of Technology, Wuhan 430068, China; 2 Department of Computer Science and Electrical Engineering, West Virginia University, Morgantown, WV 26506, USA

**Keywords:** perovskite light-emitting diodes, three-step spin coating, hole transport layer, PEDOT:PSS/MoO_3_-ammonia composite

## Abstract

High efficiency perovskite light-emitting diodes (PeLEDs) using PEDOT:PSS/MoO_3_-ammonia composite hole transport layers (HTLs) with different MoO_3_-ammonia ratios were prepared and characterized. For PeLEDs with one-step spin-coated CH_3_NH_3_PbBr_3_ emitter, an optimal MoO_3_-ammonia volume ratio (0.02) in PEDOT:PSS/MoO_3_-ammonia composite HTL presented a maximum luminance of 1082 cd/m^2^ and maximum current efficiency of 0.7 cd/A, which are 82% and 94% higher than those of the control device using pure PEDOT:PSS HTL respectively. It can be explained by that the optimized amount of MoO_3_-ammonia in the composite HTLs cannot only facilitate hole injection into CH_3_NH_3_PbBr_3_ through reducing the contact barrier, but also suppress the exciton quenching at the HTL/CH_3_NH_3_PbBr_3_ interface. Three-step spin coating method was further used to obtain uniform and dense CH_3_NH_3_PbBr_3_ films, which lead to a maximum luminance of 5044 cd/m^2^ and maximum current efficiency of 3.12 cd/A, showing enhancement of 750% and 767% compared with the control device respectively. The significantly improved efficiency of PeLEDs using three-step spin-coated CH_3_NH_3_PbBr_3_ film and an optimum PEDOT:PSS/MoO_3_-ammonia composite HTL can be explained by the enhanced carrier recombination through better hole injection and film morphology optimization, as well as the reduced exciton quenching at HTL/CH_3_NH_3_PbBr_3_ interface. These results present a promising strategy for the device engineering of high efficiency PeLEDs.

## 1. Introduction

Taking advantage of high photoluminescence quantum yield (PLQY), excellent color purity, high carrier mobility and low-temperature solution-processing, organometal halide perovskites have been studied extensively for their applications in solution-processed light-emitting diodes (LEDs) [1,2]. Since Friend’s group reported the first demonstration about room-temperature infrared and green light emission observed in LEDs with CH_3_NH_3_PbX_3_ (X is I^−^, Br^-^ or Cl^−^) perovskite emission layers (EML) in 2014 [3], organic-inorganic perovskite light-emitting diodes (PeLEDs) have attracted much attention and their external quantum efficiency (EQE) of PeLEDs increased rapidly from 0.1% to exceeding 20% [3,4,5,6,7]. Although possessing the excellent EQE comparable with quantum dot light-emitting diodes (QLEDs) and organic light-emitting diodes (OLEDs), PeLEDs still face challenges for the commercial application, such as device performance and stability.

In PeLEDs, poly(styrenesulfonate)-doped poly(3,4-ethylenedioxythiophene) (PEDOT:PSS) is the most frequently selected hole transport material (HTM), which can reduce the surface roughness of indium tin oxide (ITO) and the energy barrier between ITO and perovskite materials. However, it can erode the ITO substrate because of its acidic nature and finally affect the performance and reliability of devices [8]. Although PEDOT:PSS possesses lowest unoccupied molecular orbital (LUMO) of 5.2 eV, which are beneficial for the hole injection and transport, exciton quenching usually takes place at PEDOT:PSS/perovskite interface [9]. Therefore, further modification of PEDOT:PSS is still required for the improved performance of PeLEDs.

Different methods have been used to modify the PEDOT:PSS layer and prevent exciton quenching at PEDOT:PSS/perovskite interface. Cho et al. constructed a PEDOT:PSS/perfluorinated ionomer (PFI) composite layer to adjust the work function of HTL in PeLEDs, which leads to a reduced hole injection barrier and balanced injection of charge carriers. Meanwhile, the exciton quenching at the PEDOT:PSS/CH_3_NH_3_PbBr_3_ interface could be suppressed by increasing the PFI quantity in HTL [4,5]. Besides, transition metal oxides (TMOs) have attracted much attention because of their excellent properties such as high transparency, tunable morphology, and good electrical conductivities. TMOs, such as MoO_3_ [10,11], WO_3_ [12], and V_2_O_5_ [13], are stable p-type semiconductor materials, which is promising to substitute or modify PEDOT:PSS. MoO_3_, which has high work function, is one of the most studied oxide HTMs used in both LEDs and organic solar cells [14,15]. In detail, MoO_3_ has been used as interlayers to enhance the hole injection or transport in OLEDs [14,15,16], organic solar cells (OPVs) [17,18,19,20,21], and perovskite solar cells [22,23]. However, it has been less employed to modify HTL such as PEDOT:PSS to obtain the improved performance of OLEDs and PeLEDs, not only through balanced carrier transport, but also by suppressing the exciton quenching.

Several groups have tried to employ the MoO_3_ doped HTLs in organic-inorganic PeLEDs and other LEDs in order to obtain better performance of devices [24,25,26]. Kim et al. [25] reported the enhanced performance of CH_3_NH_3_PbBr_3_ PeLEDs caused by a solution-processed MoO_3_ and PEDOT:PSS (PEDOT:MoO_3_) composite HTL with the MoO_3_ concentration in the range of 0.1~0.7 wt.%. The hole injection was improved by doping MoO_3_ in PEDOT:PSS through a reduction in the contact barrier at HTL/CH_3_NH_3_PbBr_3_ interface and enhanced crystallinity of perovskite film. It is noted that the MoO_3_ concentration in the composite is small, and the electron transport material (SPW-111) is not usually used in PeLEDs. Besides, Zheng et al. developed a composite hole injection layer (HIL) of MoO*_x_*-doped GO in tris(8-hydroxy-quinolinato)aluminum (Alq_3_)-based OLEDs [24], and Meng et al. modified the PEDOT:PSS HTL by doping a MoO_3_ ammonia solution with largely adjusted volume ratio of (0~0.8):1 in all inorganic CsPbBr_3_ PeLEDs [26]. According to these results, the doping of MoO_3_ in HTLs is promising to reduce the contact barrier and luminescent quenching at PEDOT:PSS/EML interface. Although a few results have been reported as regards PeLEDs with MoO_3_ doped HTLs, there is still great room for an improvement in terms of device’s efficiency and stability, and the elucidation of corresponding physical mechanism.

In this paper, PEDOT:PSS/MoO_3_-ammonia composite HTLs with different MoO_3_-ammonia ratios were introduced in organic-inorganic PeLEDs with a simple structure of ITO/HTL/CH_3_NH_3_PbBr_3_/TPBi/LiF/Al in order to suppress the exciton quenching and reduce the contact barrier at HTL/EML interface, facilitating the balanced transport of carriers. Three-step spin coating method was also employed to obtain uniform and dense CH_3_NH_3_PbBr_3_ films, which lead to a maximum luminance of 5044 cd/m^2^ and maximum current efficiency of 3.12 cd/A, showing enhancement of 750% and 767% compared with the control device respectively.

## 2. Experimental Section

### 2.1. Materials

PEDOT:PSS (Clevios P AI4083), Molybdenum trioxide (MoO_3_ powder) and Ammonium hydroxide aqueous solution were purchased from Heraeus Materials Technology Co., Ltd. (Hanau, Germany), Shanghai Aladdin Bio-Chem Technology Co., Ltd. (Shanghai, China), and Sinopharm Chemical Reagent Co., Ltd. (Shanghai, China) respectively. Methylammonium bromine (CH_3_NH_3_Br) was purchased from Xi’an Polymer Light Technology Co., Ltd. (Xi’an, China). Lead Bromide (PbBr_2_, 99.999%) and N,N-Dimethylformamide (DMF, 99.9%) were purchased from Sigma-Aldrich Co., Ltd. (St. Louis, MO, USA). 2,2′,2′-(1,3,5-benzinetriyl)-tris(1-phenyl-1-H-benzimidazole) (TPBi) and Aluminum slug (Al, 99.999%) were purchased from Jilin Optical and Electronic Materials Co., Ltd. (Changchun, China) and Alfa Aesar (Ward Hill, MA, USA) respectively. PEDOT:PSS was filtered through a 0.45 μm PTFE filter before use, while other materials and solvents were used directly without further purification. The perovskite precursor solution was prepared by dissolving MABr and PbBr_2_ with a molar ratio of 2:1 in DMF solvent to obtain a fixed concentration of 5 wt.%. PEDOT:PSS/MoO_3_-ammonia composite HTLs with different MoO_3_-ammonia ratios were obtained by using the method referred to the literature [26]. After dissolving MoO_3_ powder in ammonium hydroxide aqueous solution to obtain 5 mg/mL MoO_3_-Ammonia mixed solution, PEDOT:PSS and MoO_3_-Ammonia solution with different volume ratios (1:0.01, 1:0.02, 1:0.03) were mixed to prepare PEDOT:PSS/MoO_3_-ammonia composite solution, which should be stirred for 1 h before use.

### 2.2. Device Fabrication

Our PeLEDs were prepared on pre-patterned ITO-coated glass substrates with the sheet resistance of ~15 Ω/m^2^. The basic device structure is ITO/composite HTL/CH_3_NH_3_PbBr_3_/TPBi/LiF/Al. Typically, the substrates were cleaned ultrasonically in acetone, methanol and deionized water for 5 min sequentially. After drying with a nitrogen gun, the substrates were treated by oxygen plasma for 5 min in order to modify the work function of ITO effectively.

Next, these substrates were moved into a glovebox to spin-coat pure PEDOT:PSS, PEDOT:PSS/MoO_3_-ammonia composite and CH_3_NH_3_PbBr_3_ layers. The PEDOT:PSS and PEDOT:PSS/MoO_3_-ammonia composite layers were spin-coated onto the substrates at 8000 rpm for 30 s, and then annealed at 150 °C for 15 min in a nitrogen atmosphere. For the one-step spin coating, the perovskite precursor solution was spin-coated at 8000 rpm for 30 s, and then annealed at 80 °C for 10 min. While for the three-step spin coating, the precursor was spin-coated by three times with sequential speeds of 2000, 4000 and 6000 rpm for 30 s, followed by annealing at 80 °C for 10 min for each-step spin coating. No anti-solvent and other additives were used in the spin coating of CH_3_NH_3_PbBr_3_ layers.

Finally, the substrates were transferred to a physical vapor thermal evaporation system, in which a 30 nm TPBi, a 0.5 nm LiF and a 100 nm Al were deposited sequentially for electron transport layer (ETL), electron injection layer (EIL) and cathode in a base pressure of ~3 × 10^−7^ Torr respectively. Each substrate contains four devices with the active area of 0.1 cm^2^. All PeLEDs were encapsulated simply with cover glass slides in the glovebox and then tested immediately in ambient air.

### 2.3. Device Characterization

The thickness of PEDOT:PSS, PEDOT:PSS/MoO_3_-ammonia composite and perovskite films were recorded by an Alpha-Step D-600 stylus profiler (KLA Corporation, Milpitas, CA, USA). The absorption spectra, transmittance spectra and photoluminescence (PL) spectra were carried out with a HITACHI U-3900 ultraviolte/visible spectrophotometer and a HITACHI F-4600 luminescence spectrometer (Japan), respectively. The surface morphology of perovskite films were observed with a scanning electron microscopy (SEM, FEI Sirion FEG, FEI Corporation, Eindhoven, Netherlands). X-ray diffraction (XRD) patterns were measured with a PANalytical Empyrean X-ray diffractometer (PANalytical B. V., Almelo, Netherlands). The luminance-current density-voltage (L-J-V) characteristics of PeLEDs were tested using a Keithley 2400 source meter and a Keithley 2000 multimeter (Tektronix, Inc., Beaverton, OR, USA) coupled with a calibrated silicon photodetector (1 cm in diameter), which capture and convert photons emitted from the glass side. The electroluminescence (EL) spectra of the devices were monitored by an Ocean Optics fiber-optic spectrometer (Ocean Optics, Inc., Largo, FL, USA).

## 3. Results and Discussion

**Figure 1:**
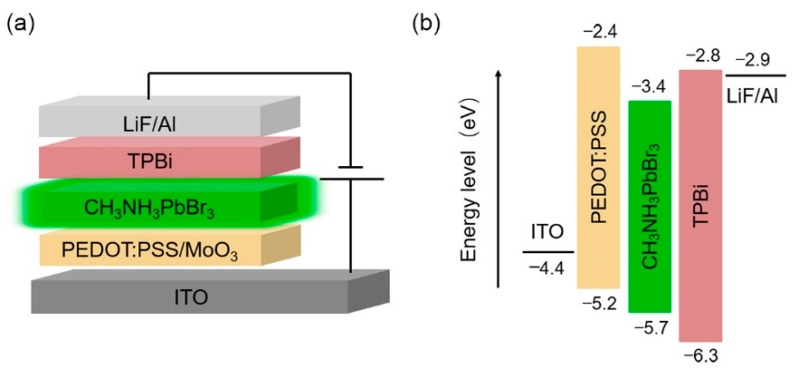
(**a**) The schematic structure of our PeLEDs. (**b**) The energy level diagram of the PeLEDs with a pure PEDOT:PSS HTL.

Figure 1a shows the schematic structure of our PeLEDs, which consist of ITO as a transparent anode, the PEDOT:PSS/MoO_3_-ammonia composite as a HTL, CH_3_NH_3_PbBr_3_ as an EML, TPBi as an ETL, LiF as an EIL, and Al as a cathode, respectively. To obtain good surface morphology of CH_3_NH_3_PbBr_3_ films, they were prepared by one-step and three-step spin coating of a CH_3_NH_3_PbBr_3_ precursor respectively. Figure 1b shows energy level diagrams of the PeLEDs with a pure PEDOT:PSS HTL. It is noted that the energy barrier between the PEDOT:PSS and CH_3_NH_3_PbBr_3_ layers is ~0.5 eV, which may result in low device efficiency. The doping of MoO_3_-ammonia in the PEDOT:PSS is expected to increase the work function of the PEDOT:PSS HTL and correspondingly reduce the contact barrier between the HTL and the CH_3_NH_3_PbBr_3_ EML for efficient hole injection [25,26].

**Figure 2:**
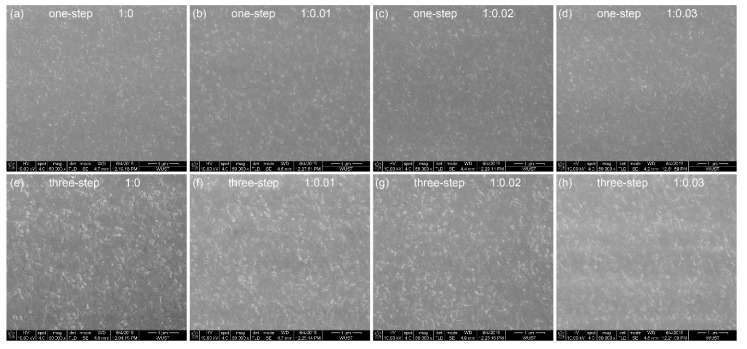
SEM images of the one-step spin-coated CH_3_NH_3_PbBr_3_ film samples on (**a**) PEDOT:PSS, (**b**) PEDOT:PSS/MoO_3_-ammonia (1:0.01), (**c**) PEDOT:PSS/MoO_3_-ammonia (1:0.02), (**d**) PEDOT:PSS/MoO_3_-ammonia (1:0.03) composite HTLs, and three-step spin-coated CH_3_NH_3_PbBr_3_ film samples on (**e**) PEDOT:PSS, (**f**) PEDOT:PSS/MoO_3_-ammonia (1:0.01), (**g**) PEDOT:PSS/MoO_3_-ammonia (1:0.02), (**h**) PEDOT:PSS/MoO_3_-ammonia (1:0.03) composite HTLs.

Figure 2 shows the SEM images of CH_3_NH_3_PbBr_3_ films prepared on pure PEDOT:PSS and PEDOT:PSS/MoO_3_-ammonia composite HTLs [glass/ITO/composite HTL (~40 nm)/CH_3_NH_3_PbBr_3_ (~30 nm for one-step spin coating, ~55 nm for three-step spin coating)], respectively. The effect of the small MoO_3_-ammonia amount on the surface morphology of CH_3_NH_3_PbBr_3_ film is not evident. As reported in the literature, multi-step spin coating is expected to improve the surface morphology of perovskite film [27,28]. As shown in Figure 2, uniform and compact perovskite films with enhanced crystallinity formed on increasing the coating times from one to three.

**Figure 3:**
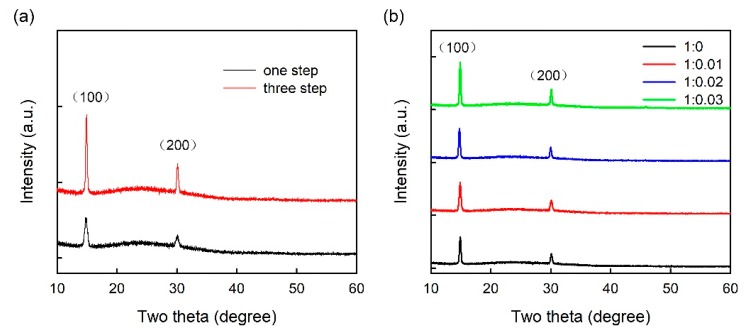
XRD patterns of (**a**) one-step and three-step coated CH_3_NH_3_PbBr_3_ films on the pure PEDOT:PSS film, (**b**) three-step coated CH_3_NH_3_PbBr_3_ films on the pure PEDOT:PSS film and PEDOT:PSS/MoO_3_-ammonia composite HTLs with different MoO_3_ ratios. The XRD patters were shifted vertically for clarity.

Figure 3 shows XRD patterns of one-step and three-step coated CH_3_NH_3_PbBr_3_ films (glass/composite HTL (~40 nm)/CH_3_NH_3_PbBr_3_ (~30 nm for one-step spin coating, ~55 nm for three-step spin coating)). All the XRD patterns show two characteristic peaks at 15° and 30°, assigned to (100) and (200) crystal planes respectively, suggesting the crystal growth orientation along (100) planes. As shown in Figure 3a, the intensity of diffraction peaks was enhanced in three-step spin-coated CH_3_NH_3_PbBr_3_ film compared with one-step spin-coated CH_3_NH_3_PbBr_3_ film on pure PEDOT:PSS film, suggesting a better crystallization on increasing the coating time from one to three. As shown in Figure 3b, on increasing the ratio of MoO_3_-ammonia from 0 to 0.03, the intensity of diffraction peaks of three-step spin-coated CH_3_NH_3_PbBr_3_ film increases monotonically. The similar trend is also found in the one-step spin-coated CH_3_NH_3_PbBr_3_ films with different MoO_3_-ammonia ratios. This result is well consistent with previously reported results [25,26], which may be explained by that the MoO_3_ particles can act as crystal nuclei for the growth of spin-coated perovskite film.

**Figure 4:**
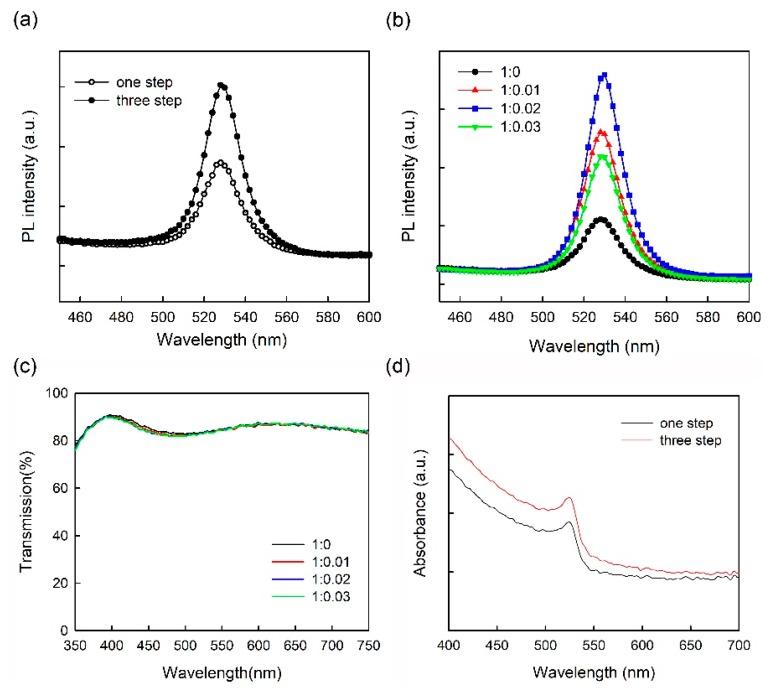
PL spectra of (**a**) one-step and three-step coated CH_3_NH_3_PbBr_3_ films on the pure PEDOT:PSS film, (**b**) three-step coated CH_3_NH_3_PbBr_3_ films on the pure PEDOT:PSS film and PEDOT:PSS/MoO_3_-ammonia composite HTLs with different MoO_3_-ammonia ratios. (**c**) The transmittance spectra of the pure PEDOT:PSS film and PEDOT:PSS/MoO_3_-ammonia composite HTLs with different MoO_3_-ammonia ratios. (**d**) The absorption spectra of one-step and three-step spin-coated perovskite films on pure PEDOT:PSS HTL.

Figure 4 shows steady-state PL spectra of one-step and three-step coated CH_3_NH_3_PbBr_3_ films [glass/ITO/composite HTL (~40 nm)/CH_3_NH_3_PbBr_3_ (~30 nm for one-step spin coating, ~55 nm for three-step spin coating)] conducted by using a luminescence spectrometer with an excitation wavelength of 315 nm. All the PL spectra show a well-defined peak at ~528 nm. As shown in Figure 4a, PL intensity was enhanced in three-step spin-coated CH_3_NH_3_PbBr_3_ film compared with one-step spin-coated CH_3_NH_3_PbBr_3_ film on pure PEDOT:PSS film. It can be explained by that the amount and morphology of perovskite material affect the PL intensity, namely more excitons will be generated by increasing the amount of perovskite particles, leading to the enhancement of the PL intensity when the thickness of perovskite film increases from ~30 nm (one-step coating) to ~55 nm (three-step coating) shown in Figure 4a. As shown in Figure 4b, on increasing the ratio of MoO_3_-ammonia from 0 to 0.02, the PL intensity of three-step spin-coated CH_3_NH_3_PbBr_3_ film gradually increases, while the further increase of the amount of MoO_3_-ammonia leads to the decrease of the PL intensity. The similar trend is also found in the one-step spin-coated CH_3_NH_3_PbBr_3_ films with different MoO_3_-ammonia ratios. It is suggested that the optimal MoO_3_-ammonia ratio is beneficial for blocking the exciton quenching at the HTL/CH_3_NH_3_PbBr_3_ interface, while the excessive MoO_3_-ammonia ratio is unfavorable. These results may be due to the increase of MoO_3_ on top of the HIL separating excitons generated in the CH_3_NH_3_PbBr_3_ EML from the quenching of PEDOT:PSS. However, on increasing the MoO_3_-ammonia amount, dopant aggregation or trap states may also occur at the HTL/EML interface, leading to the decay of photoluminescence. Figure 4c shows the transmittance spectra of PEDOT:PSS and PEDOT:PSS/MoO_3_-ammonia composite films with different MoO_3_-ammonia ratios (glass/ITO/composite HTL (~40 nm)). As shown in transmittance spectra, a small amount of MoO_3_ has little effect on the transmittance of PEDOT:PSS/MoO_3_-ammonia composite films in the visible range. The transmittances of four samples are near-identical, indicating that the doping of MoO_3_-ammonia in PEDOT:PSS HTL cannot impede the light passing through the HTL in this work. Figure 4d shows the absorption spectra of one-step and three-step spin-coated perovskite films on pure PEDOT:PSS HTL. Both two absorption spectra show a well-defined peak at ~526 nm. Furthermore, the absorption intensity was enhanced on increasing the coating time from one to three, which can be attributed to the increase of the thickness of perovskite film from ~30 nm (one-step coating) to ~55 nm (three-step coating).

**Figure 5:**
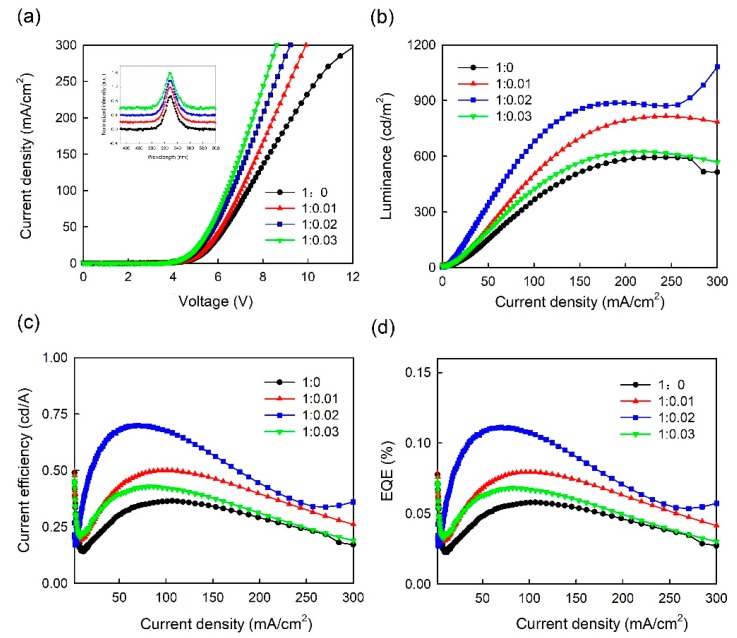
(**a**) J-V, (**b**) L-J, (**c**) CE-J, (**d**) EQE-J curves of PeLEDs with a one-step spin-coated emitter and a PEDOT:PSS/MoO_3_-ammonia (1:0, 1:0.01, 1:0.02, 1:0.03) composite HTL (device S1, S2, S3, S4). The inset is normalized EL spectra of the PeLED devices at 20 mA/cm^2^, which were shifted vertically for clarity.

Figure 5 shows (a) the current density vs. voltage (J-V), (b) the luminance vs. current density (L-J), (c) the current efficiency vs. current density (CE-J), and (d) the EQE vs. current density (EQE-J) curves for the single-step CH_3_NH_3_PbBr_3_ PeLEDs with pure PEDOT:PSS and PEDOT:PSS/MoO_3_-ammonia (1:0.01, 1:0.02, 1:0.03) composite HTLs. These four devices are labelled as S1, S2, S3, and S4 for clarity respectively. The detailed device parameters of the PeLEDs (S1, S2, S3, S4) are summarized in Table 1. As shown in Figure 5a, on increasing the MoO_3_-ammonia ratio from 0 to 0.03, the turn-on voltage, which are defined as the driving voltage at ∼1 mA/cm^2^, decreases monotonically from 4.3 V to 4.14 V. Besides, the current density of the PeLEDs increases on increasing the MoO_3_-ammonia amount, suggesting a reduced energy barrier at HTL/EML interface, inducing more efficient hole injection into CH_3_NH_3_PbBr_3_ layer [25,26]. As described in the L-J, CE-J, and EQE-J characteristics, a maximum luminance of 1082 cd/m^2^, a maximum CE of 0.7 cd/A and a maximum EQE of 0.11% were observed in the device with the MoO_3_-ammonia ratio of 0.02 (device S3), indicating the optimal volume ratio, while for the control device with pure PEDOT:PSS HTL (device S1), the maximum luminance of 593 cd/m^2^ and maximum CE of 0.36 cd/A were obtained. Therefore, the optimized device shows a 82% enhancement in the maximum luminance and 94% enhancement in the maximum CE respectively.

These results suggest that the hole injection in the PeLEDs with the PEDOT:PSS/MoO_3_-ammonia composite layer can be improved by reducing the contact barrier [25,26] and blocking the exciton quenching at the HTL/CH_3_NH_3_PbBr_3_ interface [26]. However, the device efficiency decreases when an excessive MoO_3_-ammonia amount was doped in the PEDOT:PSS/MoO_3_-ammonia composite HTL (0.03), possibly due to the trap states formed at HTL/EML interface after the doping of the excessive MoO_3_-ammonia. The inset in Figure 5a shows the normalized electroluminescence (EL) spectra of CH_3_NH_3_PbBr_3_ PeLEDs using PEDOT:PSS/MoO_3_-ammonia composite HTLs with different amounts of MoO_3_-ammonia, indicating that the EL spectra of PeLEDs with composite HTLs (S2, S3, S4) are nearly identical to that with pure PEDOT:PSS HTL (S1). It is suggested that the MoO_3_-ammonia doping cannot modify the emission profiles of CH_3_NH_3_PbBr_3_ PeLEDs, which have an EL peak at ∼528 nm.

**Figure 6:**
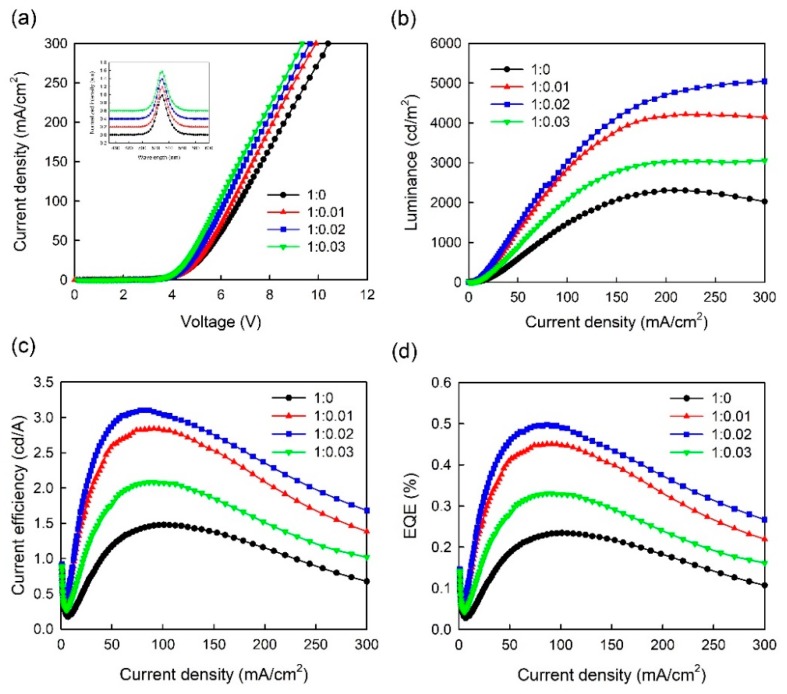
(**a**) J-V, (**b**) L-J, (**c**) CE-J, (**d**) EQE-J curves of PeLEDs with a three-step spin-coated emitter and a PEDOT:PSS/MoO_3_-ammonia (1:0, 1:0.01, 1:0.02, 1:0.03) composite HTL (device T1, T2, T3, T4). The inset is normalized EL spectra of the PeLED devices at 20 mA/cm^2^, which were shifted vertically for clarity.

**Figure 7:**
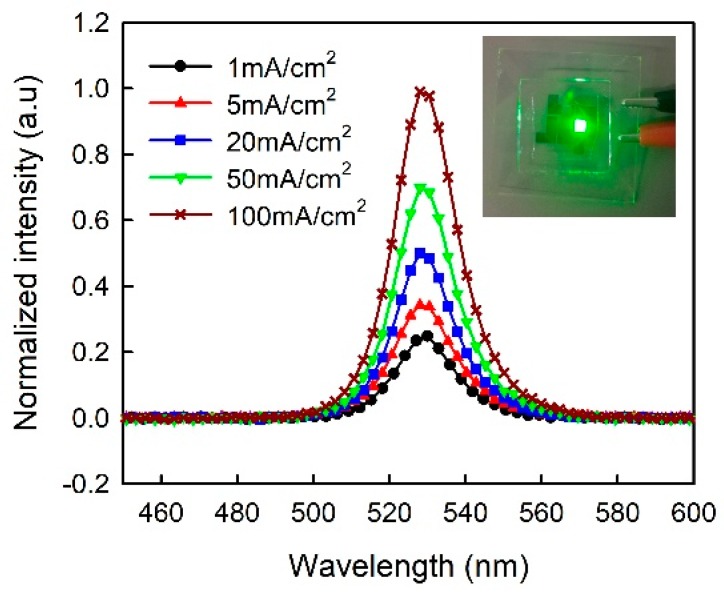
EL spectra of the PeLED devices with a three-step spin-coated emitter and a PEDOT:PSS/MoO_3_-ammonia (1:0.02) composite HTL at different current densities. The inset is a luminescence image of the device at 50 mA/cm^2^.

Figure 6 shows J-V, L-J, CE-J, and EQE-J curves for the three-step CH_3_NH_3_PbBr_3_ PeLEDs with pure PEDOT:PSS and PEDOT:PSS/MoO_3_-ammonia (1:0.01, 1:0.02, 1:0.03) composite HTLs, which are labelled as T1, T2, T3, and T4 for clarity respectively. The detailed device parameters of the PeLEDs (T1, T2, T3, T4) are summarized in Table 2. As shown in Figure 6a, on increasing the MoO_3_-ammonia ratio from 0 to 0.03, the turn-on voltage decreases from 4.08 V to 3.68 V. Besides, the current density of the PeLEDs increases on increasing the MoO_3_-ammonia amount, which is similar to the trend observed in one-step devices (S1, S2, S3, S4). As shown in Figure 6c,d, the CE and EQE increase at low current densities because of the rapidly increased luminance (or the number of excitons). On further increasing the current density, the luminance increases more slowly or decreases, leading to the decreased CE and EQE, namely the efficiency roll-off. As reported, the efficiency roll-off of OLEDs is mainly caused by charge imbalance and quenching processes [10,29,30]. Similarly, in this work, higher CE found in the device with the MoO_3_-ammonia ratio of 0.02 can be explained by the balance of electrons and holes in the EM, as well as the reduced exciton quenching. From the L-J, CE-J, and EQE-J characteristics, a maximum luminance of 5044 cd/m^2^, a maximum CE of 3.12 cd/A and a maximum EQE of 0.5% were also observed in the device with the optimal MoO_3_-ammonia ratio of 0.02 (device T3), while for the device with pure PEDOT:PSS HTL (device T1), the maximum luminance of 2309 cd/m^2^ and maximum CE of 1.47 cd/A were obtained. Thus, the optimized device shows a 118% enhancement in the maximum luminance and 112% enhancement in the maximum CE respectively. Compared with the control device with pure PEDOT:PSS HTL and one-step spin-coated CH_3_NH_3_PbBr_3_ film (device S1), a 750% enhancement in the maximum luminance and 767% enhancement in the maximum CE were obtained for the optimized device (device T3). The inset in Figure 6a shows the normalized EL spectra of three-step CH_3_NH_3_PbBr_3_ PeLEDs using PEDOT:PSS/MoO_3_-ammonia composite HTLs with different amounts of MoO_3_-ammonia, in which all PeLEDs have an EL peak at ∼528 nm. Figure 7 shows the EL curves measured at different current densities. The results indicate that all PeLEDs have an EL peak at ~528 nm, suggesting the color stability of our devices.

These results indicate that the hole injection in the PeLEDs with the PEDOT:PSS/MoO_3_-ammonia composite layer can be improved by reducing the contact barrier [25,26] and suppressing the exciton quenching at the HTL/CH_3_NH_3_PbBr_3_ interface [26]. Besides, three-step spin coating method can improve the surface morphology of the CH_3_NH_3_PbBr_3_ perovskite film shown in Figure 2. Furthermore, the as-obtained perovskite layer exhibited a stronger PL intensity shown in Figure 4. These factors induce the significant improvement on luminous performance of our PeLEDs. Therefore, the significantly improved efficiency of PeLEDs using three-step spin-coated CH_3_NH_3_PbBr_3_ film and an optimum PEDOT:PSS/MoO_3_-ammonia composite HTL can be explained by the enhanced carrier recombination through better hole injection and film morphology optimization, as well as the reduced exciton quenching at HTL/CH_3_NH_3_PbBr_3_ interface.

## 4. Conclusions

In summary, we demonstrated improved performance of PeLEDs using a PEDOT:PSS/MoO_3_-ammonia composite HTL by reducing the energy barrier and blocking the exciton quenching at the HTL/CH_3_NH_3_PbBr_3_ interface. For PeLEDs with one-step spin-coated CH_3_NH_3_PbBr_3_ film, an enhancement of 82% in the maximum luminance and 94% in the maximum CE was found in PeLED with an optimal MoO_3_-ammonia volume ratio (0.02) in PEDOT:PSS/MoO_3_-ammonia composite HTL compared with the control device with pure PEDOT:PSS HTL respectively. Three-step spin coating method was further used to obtain uniform and dense CH_3_NH_3_PbBr_3_ films, which lead to a maximum luminance of 5044 cd/m^2^ and maximum CE of 3.12 cd/A, which are 750% and 767% larger than those of the control device respectively. The significantly improved efficiency of PeLEDs using three-step spin-coated CH_3_NH_3_PbBr_3_ film and an optimum PEDOT:PSS/MoO_3_-ammonia composite HTL can be originated from the enhanced carrier recombination through better hole injection and film morphology optimization, as well as the reduced exciton quenching at HTL/CH_3_NH_3_PbBr_3_ interface. These results suggest a promising clue for the device engineering of high efficiency PeLEDs.

## Figures and Tables

**Table 1:** Summary of the device parameters of PeLEDs (device S1, S2, S3, S4) with a single-step spin-coated emitter and a PEDOT:PSS/MoO_3_-ammonia (1:0, 1:0.01, 1:0,02, 1:0.03) composite HTL.

Volume Ratio of PEDOT:PSS/MoO_3_-Ammonia	L_max_ (cd/m^2^)	CE_max_ (cd/A)	EQE_max_ (%)	Turn-on Voltage (V)
1:0 (S1)	593	0.36	0.057	4.3
1:0.01 (S2)	816	0.5	0.079	4.22
1:0.02 (S3)	1082	0.7	0.11	4.18
1:0.03 (S4)	624	0.42	0.068	4.14

**Table 2:** Summary of the device parameters of PeLEDs (device T1, T2, T3, T4) with a three-step spin-coated emitter and a PEDOT:PSS/MoO_3_-ammonia (1:0, 1:0.01, 1:0,02, 1:0.03) composite HTL.

Volume Ratio of PEDOT:PSS/MoO_3_-Ammonia	L_max_ (cd/m^2^)	CE_max_ (cd/A)	EQE_max_ (%)	Turn-on Voltage (V)
1:0 (T1)	2309	1.47	0.23	4.08
1:0.01 (T2)	4215	2.84	0.45	3.84
1:0.02 (T3)	5044	3.12	0.5	3.75
1:0.03 (T4)	3055	2.08	0.33	3.68
